# Mechanical Properties, Corrosion Behavior, and In Vitro Cell Studies of the New Ti-25Ta-25Nb-5Sn Alloy

**DOI:** 10.3390/ma16051970

**Published:** 2023-02-28

**Authors:** Kerolene Barboza da Silva, João Pedro Aquiles Carobolante, S. Sudhagara Rajan, Celso Bortolini Júnior, Roberta Maia Sabino, Maurício Rangel Seixas, Roberto Zenhei Nakazato, Ketul C. Popat, Ana Paula Rosifini Alves Claro

**Affiliations:** 1 School of Engineering, São Paulo State University (Unesp), Ilha Solteira 15385-000, Brazil; 2 Department of Materials and Technology, School of Engineering and Sciences, São Paulo State University (Unesp), Guaratinguetá 12516-410, Brazil; 3 School of Advanced Materials Discovery, Colorado State University (CSU), Fort Collins, CO 80523, USA; 4 Department of Chemistry and Energy, School of Engineering and Sciences, São Paulo State University (Unesp), Guaratinguetá 12516-410, Brazil; 5 Department of Mechanical Engineering, School of Biomedical Engineering, Colorado State University (CSU), Fort Collins, CO 80523, USA

**Keywords:** biomaterials, Ti-Ta-Nb-Sn, cell culture, corrosion behavior, mechanical properties

## Abstract

This study aims to characterize a new Ti-25Ta-25Nb-5Sn alloy for biomedical application. Microstructure, phase formation, mechanical and corrosion properties, along with the cell culture study of the Ti-25Ta-25Nb alloy with Sn content 5 mass% are presented in this article. The experimental alloy was processed in an arc melting furnace, cold worked, and heat treated. For characterization, optical microscopy, X-ray diffraction, microhardness, and Young’s modulus measurements were employed. Corrosion behavior was also evaluated using open-circuit potential (OCP) and potentiodynamic polarization. In vitro studies with human ADSCs were performed to investigate cell viability, adhesion, proliferation, and differentiation. Comparison among the mechanical properties observed in other metal alloy systems, including CP Ti, Ti-25Ta-25Nb, and Ti-25Ta-25-Nb-3Sn showed an increase in microhardness and a decrease in the Young’s modulus when compared to CP Ti. The potentiodynamic polarization tests indicated that the corrosion resistance of the Ti-25Ta-25Nb-5Sn alloy was similar to CP Ti and the experiments in vitro demonstrated great interactions between the alloy surface and cells in terms of adhesion, proliferation, and differentiation. Therefore, this alloy presents potential for biomedical applications with properties required for good performance.

## 1. Introduction

In the world, about 90% of people over the age of 40 suffer due to bone degenerative diseases, and the demand for new biomaterials that can efficiently replace body parts has increased considerably [1]. Achieving better biocompatibility and mechanical properties similar to bone is the driving force for the research and development of new alloys.

Titanium and its alloys are considered a better alternative than other metallic biomaterials because of their excellent biocompatibility, corrosion resistance, and good mechanical properties such as lower Young’s modulus and higher specific strength, toughness, and hardness [2,3]. Commercially pure titanium (CP Ti) and the Ti-6Al-4V alloy are widely used for dental and maxillofacial implants, hip and knee prostheses, and fixing devices (i.e., plates, screws, nails, and wires), among others [4]. However, inferior tensile properties and wear behavior have limited the use of the CP Ti (1), and Al and V ions released from the Ti-6Al-4V alloy have been associated with neuropathy and Alzheimer’s disease after a longer period of implantation [2,5]. Furthermore, the Young’s modulus of the CP Ti (105 GPa) and Ti-6Al-4V alloys (112 GPa) are significantly higher than the typical bone modulus (4–30 GPa), which can result in the stress shielding effect [2]. Due to this effect, the implant takes up all the stress and surrounding/adjacent bone is left with inadequate stress, causing implant loosening and failure. In some cases, is necessary revision surgery. This scenario is the impetus for researchers to develop low Young’s modulus Ti alloys with non-toxic and non-allergenic alloying elements.

Ti-based alloys with beta phase are considered an excellent alternative to the alpha- and alpha/beta-type alloys [6]. Adding elements such as Ta, Mo, Nb, Sn, and Zr have resulted in beta-type Ti alloys with superior properties and are considered suitable for biomedical applications. Ti-10Mo-8Nb [7], Ti-35Nb-5Ta-7Zr [8], Ti-35Nb-7Ta [8], Ti-13Nb-13Zr [9], and Ti-25Ta-25Nb [10,11] are examples of alloys studied.

Among these beta-type Ti alloys, the composition Ti-25Ta-25Nb possesses a low Young’s modulus and is considered more biocompatible than Ti-6Al-4V alloy due to the absence of non-toxic elements [12,13]. However, Ti-25Ta-25Nb alloy can exhibit double-yielding phenomena [14] attributed to the presence of the martensitic phase [15]. Double yielding is unfavorable for long-term biomedical applications because to stabilize the beta phase and to suppress the formation of new phases, the addition of Sn in the Ti-Ta-Nb system has been considered [16].

In the human body, besides inhibiting cancer cells, the Sn exhibits good biocompatibility and stimulates the production of protein and nucleic acids [17]. Sn has been an essential alloying element in biodegradable alloys with magnesium and zinc [17,18]. Similarly, Sn has been chosen as an alloying element with tantalum, niobium, and zirconium in titanium alloys. Ti-Nb-Sn [18,19] and Ti-Nb-Zr-Ta-Sn [20] system alloys exhibited good biocompatibility, lower Young’s modulus, and higher mechanical strength and ductility when compared to cp Ti and Ti-6Al-4V alloy. In vitro studies performed with the Ti-Nb-Sn system with the addition of Mg indicated more remarkable similarity of the properties with the bone tissue because there was increased adhesion and osteogenic differentiation of bone marrow mesenchymal stem cells (BMMSC’s) [18]. Moreover, the oxide formed from Sn has a bactericidal action [19].

Hence, a new alloy for the Ti-25Ta-25Nb system was developed by addition of 5% of Sn as an alloying element. The Ti-25Ta-25Nb-5Sn alloy’s microstructural and mechanical properties along with its corrosion behavior and cell responses were analyzed and reported in the present study.

## 2. **Materials and Methods**

### 2.1. Ti-25Ta-25Nb-5Sn Alloy Preparation

The alloy was prepared by an arc melting furnace under an argon atmosphere from commercially pure Ti (grade 2), Ta (99.99%), Nb, and Sn (99.9%) according to the studied composition (Ti-25Ta-25Nb-5Sn wt%). The ingots were remelted at least five times to ensure chemical homogeneity. After, they were homogenized at 1000 °C for 86.4 ks, cold worked (room temperature) by swaging to obtain a nearly 50% reduction in thickness, and solubilized at 950 °C for 10.8 ks followed by quenching in water.

### 2.2. Microstructural and Mechanical Characterization

Samples into discs 3 mm thick and 10 mm in diameter were cut from the ingots after each processing route steps. Phase constitutions of as-cast, homogenized, and quenched samples were investigated by X-ray diffraction (XRD Bruker D8 advance) using Cu Kα radiation operating at 40 kV and 25 mA, scanning from 30° to 100° with each step 0.02° holding for 0.35 s. Phase quantitative analysis was determined by Rietveld’s method using the Total Pattern Analysis Solution (TOPAS) software with crystallographic sheets from ICSD database. The microstructure of the Ti-25Ta-25Nb-5Sn alloy processed (after quenching) was examined by optical microscopy (Epiphot, Nikon, Tokyo, Japan). Sample was grounded with SiC papers up to 1500 grid, polished with colloidal silica, ultrasonically cleaned with deionized water and ethyl alcohol, and finally etched with Kroll’s reagent (5% HF, 10% HNO_3_ and 85% H_2_O).

Mechanical characterization was based on microhardness and Young’s modulus evaluation of the alloy after processing (quenched samples). The microhardness test was conducted at room temperature by a Vickers microindentation tester (401 MVD, Wilson Instruments, Campbellford, ON, Canada). The measures were carried out under a load of 200 g and dwell time of 10 s. Twelve indentations for each sample were performed, and the mean values are reported. The Young’s modulus was obtained by the excitation impulse method with Sonelastic equipment (ATCP) at room temperature following ASTM E1876 standard. The average values were calculated from five measurements.

### 2.3. Electrochemical *Measurements*

Open-circuit potential (OCP) and potentiodynamic polarization were used to evaluate the corrosion behavior of the Ti-25Ta-25Nb-5Sn alloy in fluorinated physiological serum (0.15 M NaCl + 0.03 M NaF) a temperature of 37 °C and pH of 6.0. The tests were carried out in a conventional three-electrode cell using a platinum electrode as counter electrode, saturated calomel electrode (SCE) as reference electrode, and quenched samples with 0.5 cm^2^ exposed area as working electrode. The OCP measurement was maintained up to 10.8 ks after the samples were immersed into the electrolyte. When the OCP became stable, potentiodynamic polarization tests were started. Potentiodynamic curves were recorded from −0.3 V against OCP to +3.0 V at a scanning rate of 1 mV/s. All electrochemical measures were performed in triplicate. The corrosion potential (Ecorr) and corrosion current density (icorr) were determined by the Tafel method using the Tafel Extrapolation application available for OriginPro 2016 software (OriginLab Corporation, Northampton, MA, USA).

### 2.4. In Vitro Studies

Human ADSCs (adipose-derived stem cells) were selected to evaluate the cell activity of the Ti-25Ta-25Nb-5Sn alloy after 4 and 7 days. For the biological assays, samples were cut into disks 10 mm in diameter and 3 mm in thickness, sanded up to 600 mesh, cleaned in an ultrasonic bath with deionized water, and sterilized. The cells were cultured in a growth medium composed of α-MEM media with 10% (*v*/*v*) fetal bovine serum and 1% (*v*/*v*) penicillin/streptomycin at 37 °C and under 5% CO_2_ atmosphere as described by Sabino et al. [21].

After 4 and 7 days of culture, the viability, adhesion and proliferation of cells were investigated using CellTiter-Blue assay and fluorescence microscopy. Detailed procedures are described in more detail in another report [22]. The images obtained were analyzed with the ImageJ 1.53e software to determine the quantity of ADSCs adhered to the surfaces.

The differentiation of human ADSCs cultured on the surfaces of the Ti-25Ta-25Nb-5Sn alloy was studied after 1 and 3 weeks of induced osteogenesis. The osteogenic induction medium was composed of growth media supplemented with 10^8^ M dexamethasone, 50 µg/mL ascorbic acid, and 6 mM β-glycerol phosphate (all from Sigma-Aldrich, St. Louis, MO, USA). The medium was renewed every 2 days for 3 weeks.

Total protein content and calcium concentration were determined after induced osteogenic differentiation by quantitative assays. Fluorescence microscopy and immunofluorescent staining of the cells were used to verify the presence of osteocalcin on surfaces. Using ImageJ 1.53e software, the percentage coverage area of stained osteocalcin was measured. The data were normalized to the total protein content on the surface. The analysis was previously described in Sabino et al. [21].

One-way analysis of variance (ANOVA) and Tukey’s tests with *p* < 0.05 as the level of significance were utilized for statistical analysis.

## 3. Results and Discussion

### 3.1. Alloy Characterization

**Figure 1:**
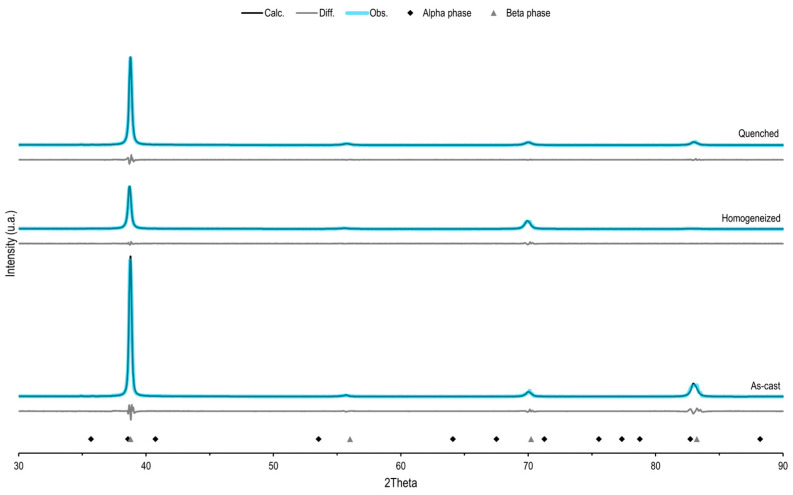
Rietveld refinement of the X-ray diffraction pattern of the Ti-25Ta-25Nb-5Sn alloy. The analysis was carried out after each processing step of the alloy: as cast, after homogenization heat treatment, and after quenching. The blue lines represent the observed XRD data. The black lines represent data calculated by Rietveld refinement. The gray lines represent the difference between the observed data and data calculated by Rietveld refinement. The diamond represents the X-ray diffraction pattern of the alpha phase, according to reference ICSD 99778. The triangle represents the X-ray diffraction pattern of the beta phase, according to reference ICSD 76165.

The structure of the Ti-25Ta-25Nb-5Sn alloy was evaluated using X-ray diffraction. Figure 1 shows the X-ray diffraction patterns observed in each step of the processing route and the pattern calculated by Rietveld refinement. Beta-indexed peaks were found in all stages of the processing route, i.e., as cast (Figure 1), after homogenization heat treatment (Figure 1), and after quenching (Figure 1). However, a low peak indexed to the alpha phase was also observed at 35.6° for the as-cast and quenched samples. The refinement of data using the Rietveld method was carried out to investigate the presence of the alpha phase. The plots and quality indicators presented in Table 1 show an adequate refinement of the data [22]. The calculated weighted residual factor (R_wp_) found in the three cases evaluated was less than 10%, and the goodness-of-fit (GOF) value was close to 1, a value obtained for ideal models. Similarly, the R_bragg_ factor, which represents the deviation between the calculated and experimental values for each phase present, was low.

Table 1 also presents the contents and lattice parameters found for the alpha and beta phases in the three processing steps evaluated. The alloying elements, Ta and Nb, cause a strong reduction in the beta transus temperature and reduce the cooling rate needed to maintain the BCC structure [23]. The influence of these alloying elements could be observed in the beta phase content found in the as cast and homogenized samples. These data show the strength of the stabilizers beta alloy elements used. However, the quenched sample (final processing condition) exhibited an increase in the alpha phase content in relation to those found in the other conditions. This goes against the expected result of the applied heat treatment, which is to stabilize the beta phase, which was already the only phase present before the quenching. Therefore, it is possible that the preparation of the sample for analysis, probably during cutting, caused the modification of a small portion of the structure to the alpha phase. Thus, considering the data obtained by XRD, the beta stabilizing strength of the alloy elements used [24,25] and previous studies [15], it is justifiable to classify the Ti-25Ta-25Nb-5Sn alloy as a metastable beta-type alloy.

The Sn content added to the alloy had no significant influence the stabilization of the structure, because there was no difference in the results found for the alloys Ti-25Ta-25Nb and Ti-25Ta-25Nb-3Sn [15]. Still, beta-type alloys with BCC structure are interesting for materials intended for application in medical devices related to bone tissue as they have more adequate mechanical properties, especially Young’s modulus lower than that found in the alpha phase [2].

According to the lattice parameters observed Table 1, the processing route led to a contraction of the unit cell, and it was expected that there was an expansion of the unit cell at the end of processing due to the heat treatments used [26]. After the homogenization heat treatment, there was an increase in the BCC unit cell lattice parameter due to the slower cooling when compared to the material in as-cast condition. With the performance of the solubilization heat treatment, an expansion of the unit cell was expected and that this expansion was maintained due to rapid cooling in the quenching, but the parameters of the BCC structure decreased, and maybe the volume occupied by the alpha-phase originated during the preparation of the samples for analysis and caused the contraction of the beta phase.

**Figure 2:**
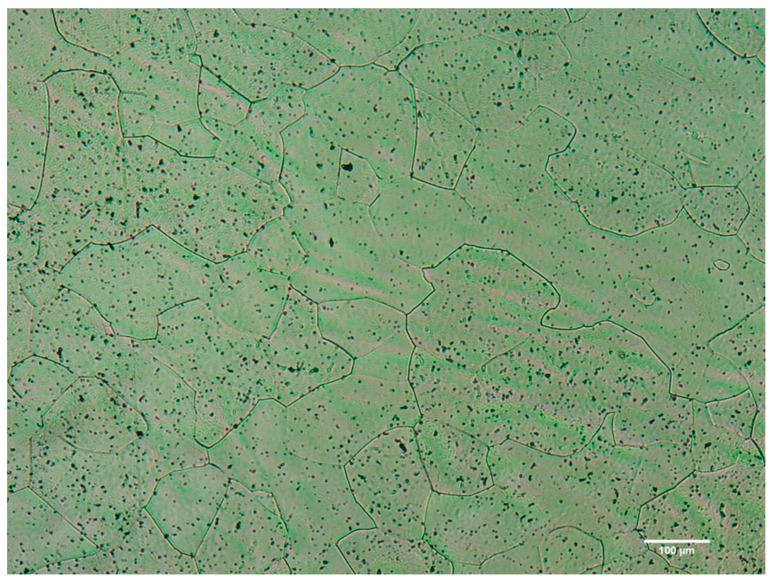
Optical micrograph of the Ti-25Ta-25Nb-5Sn alloy. Image of the microstructure of the Ti-25Ta-25Nb-5Sn after quenching.

Optical microscopy revealed (Figure 2) characteristic grains of the beta phase, reinforcing the hypothesis that the alpha phase found at the end of processing by XRD analysis originated during the preparation of samples for analysis. There is an increase in grain size concerning the ternary alloy Ti-25Ta-25Nb-3Sn evaluated in a previous study [15] relating the increase in Sn content to grain growth.

### 3.2. Mechanical Properties

**Figure 3:**
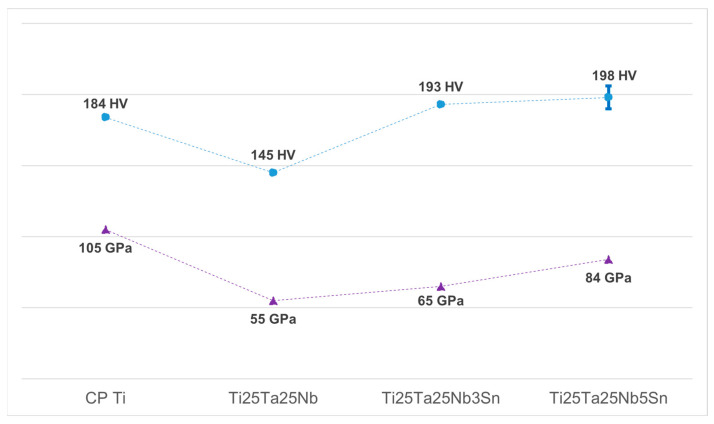
Mechanical properties of Ti-25Ta-25Nb-5Sn alloy compared with CP Ti, Ti-25Ta-25Nb and Ti-25Ta-25Nb-3Sn.

The mechanical characteristics of Ti-25Ta-25Nb-5Sn alloy obtained by Young’s modulus and microhardness measurements. Figure 3 shows a comparison among the mechanical properties of the Ti-25Ta-25Nb-5Sn, CP Ti, Ti-25Ta-25Nb-3Sn, and Ti-25Ta-25Nb alloys.

From Figure 3, it can be said that a considerably lower Young’s modulus (84 GPa) was obtained for the Ti-25Ta-25Nb-5Sn alloy compared to that of CP Ti (105 GPa) reported by Zhang and Chen [27]. This result is consistent with that of beta-type Ti alloys. According to Wei et al. [28], the Young’s modulus of Ti alloys that exhibit the beta phase is lower than that of the alpha phase since the crystalline structure is less closely packed. Several studies have shown that beta-type Ti alloys present Young’s modulus closer to human bone than commercial Ti alloys and CP Ti [29]. Depending on the form of the bone, on the direction, and on the kind of the load, the mid-thigh cortical bone has a Young’s modulus of 3–17 GPa, while the modulus of the proximal femoral trabecular is 0.441 GPa [30]. The study of the modulus is important for reducing the effect of stress shielding in orthopedic and dental implants. A similar modulus is required for long-time implants to minimize the bone atrophy caused by the stress shielding effect and to ensure the tissue’s proper regeneration [31,32,33].

On the other hand, the Young’s modulus of the studied alloy became higher in comparison with both Ti-25Ta-25Nb (55 GPa) and Ti-25Ta-25Nb-3Sn (65 GPa) alloys reported by Weng et al. [34] and Seixas et al. [15], respectively. This suggests that the increase in Sn content leads to an increase in modulus of the Ti-25Ta-25Nb ternary system alloys. As an alloying element, Sn is considered a neutral element able to stabilize the beta phase in beta-type Ti alloys; however, according to the Bo–Md diagram (d-electron based approach), the addition of Sn in the Ti-25Ta-25Nb alloy causes a decrease in Bo and Md, values indicating higher Young’s modulus [35]. Bhal et al. [36] agree that higher Sn content (>2%) can lead to increased modulus.

The Vickers microhardness values of the Ti-25Ta-25Nb-5Sn alloy (198 HV) are shown in Figure 3. It is possible to notice that this value is higher than those of the Ti-25Ta-25Nb alloy (145 HV) and CP Ti (185 HV). Bortolini Junior et al. [37] obtained a similar result for Ti-25Ta-25Nb-3Sn alloy (193 HV).

Alloying elements in different grades, heat treatments, and thermo-mechanical processing with the emergence of new phases, can manipulate the microstructures of beta-type Ti alloys and cause significant changes in their mechanical properties [38]. According to the data in Table 1, the decrease in the lattice parameter of both beta and alpha phases after the processing contributed to the increase in alloy hardness [39]. In some situations, the beta-type Ti alloys have comparable hardness with alpha- + beta-type alloys, and in others can have higher yield strength than alpha type alloys. However, the Young’s modulus of beta-type Ti alloys is significantly lower. Therefore, as a whole, the mechanical properties of β-type Ti alloys are desired [38].

### 3.3. Corrosion Behavior

**Figure 4:**
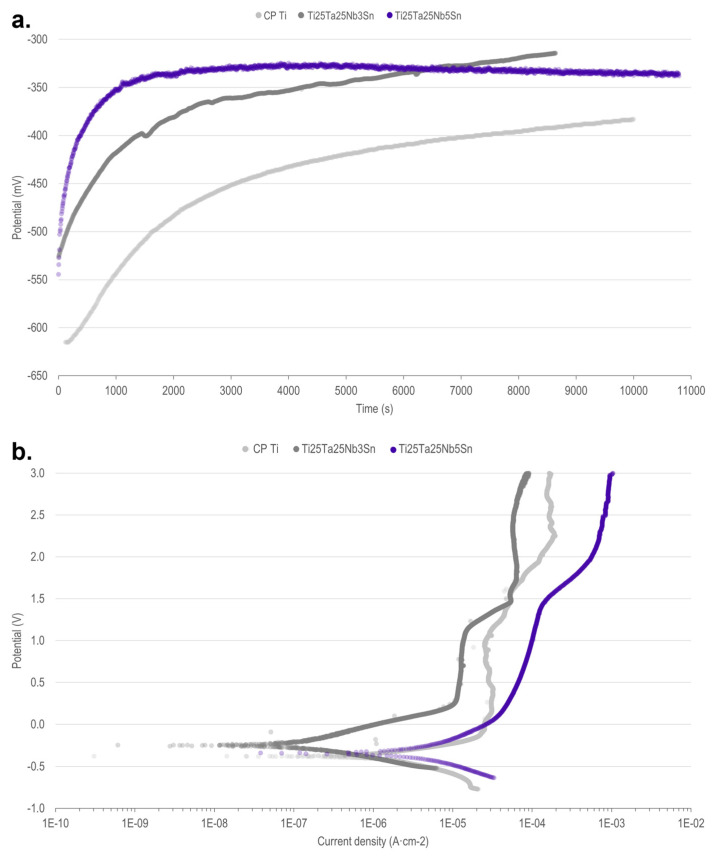
Corrosion tests of Ti-25Ta-25Nb-5Sn, Ti-25Ta-25Nb-3Sn, and CP Ti in fluoridated physiological serum (0.15 M NaCl + 0.03 M NaF (pH = 6.0)) at 37 °C. (**a**) Open-circuit potential. (**b**) Potentiodynamic polarization test.

To evaluate the corrosion resistance of the Ti-25Ta-25Nb-5Sn alloy, measurements of OCP and potentiodynamic polarization tests were performed on fluoridated physiological serum. The tests were also performed on CP Ti and Ti-25Ta-25Nb-3Sn alloy [15]. The Ti-25Ta-25Nb-5Sn alloy presented an intermediate potential value between the evaluated materials (Table 2) with a faster and clearer stabilization of the formation/dissolution of the oxide layer than that of the Ti-25Ta-25Nb-3Sn alloy and CP Ti (Figure 4a).

Figure 4b shows that the three materials evaluated form a spontaneous passive layer during the anodic phase of the electrochemical test evidenced by the stabilization of current density against the increase in the applied potential. There was a decrease in current density with the addition of Sn to Ti-25Ta-25Nb alloy [15], which is interpreted as lower corrosion resistance. Possibly, the Ta and Nb oxides formed are more chemically stable and less soluble than the Ti oxide, improving corrosion resistance [2]. When adding Sn, the oxide formed is less chemically stable and reduces corrosion resistance, reaching values close to those of CP Ti at concentrations above 5 wt% of Sn. However, the values for the alloy Ti-25Ta-25Nb-5Sn are still better than those found for CP Ti, which is suitable for biomedical applications.

### 3.4. In Vitro Studies

ADSCs were used in the evaluation of cell viability and differentiation because they are multipotent, are able to transform into osteoblasts, and due to the greater ease of obtaining other cell lines, they are a great alternative to hMSCs (human mesenchymal stem cells) [21].

**Figure 5:**
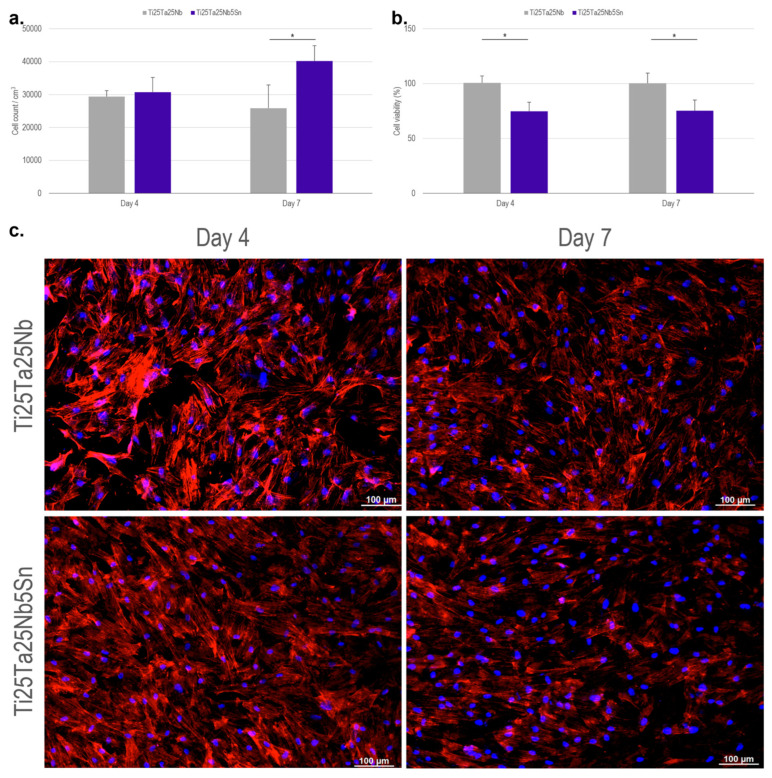
Evaluation of cell adhesion, proliferation, and cell viability on the surface of Ti-25Ta-25Nb and Ti-25Ta-25Nb-5Sn alloys. (**a**) Cell count per area, (**b**) cell viability, and (**c**) fluorescence microscopy images of ADSCs after 4 and 7 days of culture. (*) Represents the significant difference between groups, with *p* < 0.05.

Cytocompatibility was assessed based on cell counts and metabolic activity. According to Figure 5a, on the fourth day of cell culture, there was no significant difference in cell density on the surface of the Ti-25Ta-25Nb-5Sn and Ti-25Ta-25Nb alloys. There was also no significant difference between the evaluated periods, indicating that cell adhesion and proliferation reached their apex in the first days of culture. However, there was a variation in the count for the two groups on the seventh day, resulting in a significant difference. The successful implantation of a material in bone tissue is strongly dependent on the formation of bone tissue close to its surface. The initial processes of adhesion and cell proliferation are fundamental for the formation of this new bone [40,41].

Cell viability was assessed from the level of CellTiter-Blue metabolism and by fluorescence microscopy analysis (Figure 5b,c). The results for the fourth and seventh days are similar, with a significant difference between the evaluated groups. The Ti-25Ta-25Nb-5Sn alloy showed lower metabolic activity than the Ti-25Ta-25Nb alloy. However, these values were maintained during the evaluated period, suggesting that there was no regression of cellular behavior. Therefore, the viability and cell count results for Ti-25Ta-25Nb-5Sn alloy indicate potential for cell development. The cytocompatibility of titanium-based alloys with the presence of Sn has already been reported in the literature, e.g., Ti-Nb-Sn [42] and Ti-Ta-Sn [43].

**Figure 6:**
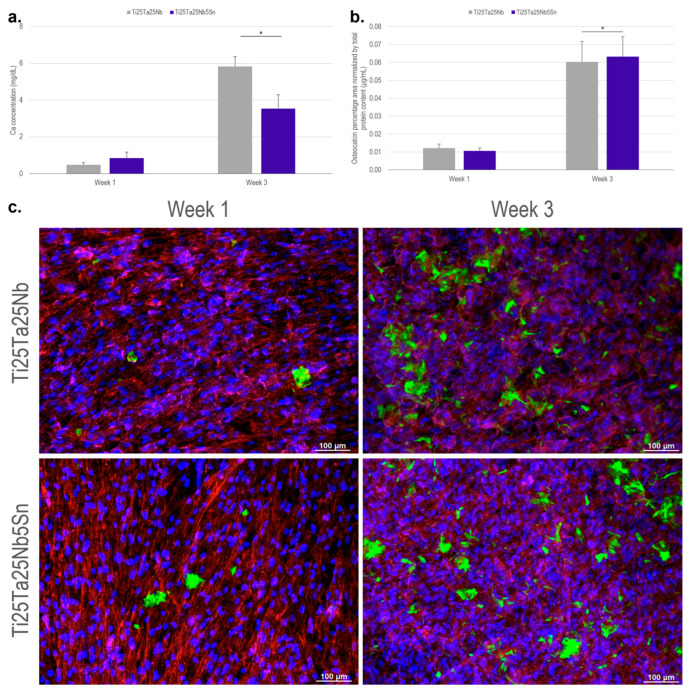
Evaluation of cell differentiation on the surface of Ti-25Ta-25Nb and Ti-25Ta-25Nb-5Sn alloys. (**a**) Ca deposited on the surfaces, (**b**) percentage area coverage by osteocalcin normalized by total protein content, and (**c**) immunofluorescence microscopy images of ADSCs after 1 and 3 weeks of induced osteogenesis. Osteocalcin is represented in green. (*) Represents the significant difference between groups, with *p* < 0.01.

For the osseointegration process to be completed, it is necessary that, in addition to being cytocompatible, the material enables osteogenesis. Therefore, after the seventh day of culture, ADSC cells were induced to differentiate into osteoblasts, and Ca and osteocalcin concentrations were evaluated. Ca is one of the mineral components of bone, so its presence indicates the occurrence of mineralization, which is one of the steps in the formation of new tissue [44]. In the third week, a significantly higher calcium concentration was observed for the Ti-25Ta-25Nb alloy (Figure 6a). However, the alloy Ti-25Ta-25Nb-5Sn also showed an increase in calcium content when evaluating the culture period.

Osteocalcin, which is produced by osteoblasts, is one of the components of the organic bone matrix and is one of the final markers of the cell differentiation process [21,45]. The osteocalcin content increased during the period evaluated (Figure 6b) as seen in the images obtained by immunofluorescent microscopy (Figure 6c). There was no significant difference between Ti-25Ta-25Nb and Ti-25Ta-25Nb-5Sn alloys. The increase in mineral deposition and organic components of bone matrix formation during the evaluated period suggests that the Ti-25Ta-25Nb-5Sn alloy can be applied in bone implants as there was no significant reduction in the cytocompatibility and differentiation results of the Ti-25Ta-25Nb alloy, and they showed similar behavior to the alloys of Ti-Nb-Sn [41] and Ti-Ta-Sn [44] systems, which present results compatible with the Ti-6Al-4V and CP Ti alloy, the main materials used in the manufacture of bone and dental implants.

## 4. Conclusions

Based on the results, it is possible to conclude that the Ti-25Ta-25Nb-5Sn alloy was obtained with beta phase formation after the processing employed. Mechanical response indicated an increase in strength and decrease of Young’s modulus in relation to CP Ti. These results have revealed the potential of the alloy for biomedical applications in bone tissue. Electrochemical studies showed the formation of an oxide layer less chemically stable that decreased corrosion resistance of the alloy, although its behavior has remained close to CP Ti. In vitro cell culture studies showed that the alloy does not have cytotoxic effects on osteoblastic cells and exhibits good biocompatibility under the analyzed conditions.

## Figures and Tables

**Table 1:** The concentrations of the phases and lattice parameters were obtained by Rietveld refinement.

Ti25Ta25Nb5Sn	R_wp_	GOF	R_Bragg_	Phase	Content (wt%)	a (Å)	c (Å)	Volume (Å^3^)
As-cast	9.92	2.46	4.959	Alpha	0.060	2.921	4.433	32.761
			0.579	Beta	99.94	3.315	-	36.415
Homogenized	6.80	1.31	1.201	Beta	100.00	3.304	-	36.304
Quenched	6.04	1.34	4.635	Alpha	1.72	2.897	4.675	33.988
			0.370	Beta	98.28	3.074	-	29.053

GOF: goodness of fit.

**Table 2:** Parameters obtained by corrosion tests for the Ti25Ta25Nb5Sn alloy compared to the CP Ti and Ti25Ta25Nb3Sn alloys. Corrosion tests were performed on fluoridated physiological serum (0.15 M NaCl + 0.03 M NaF (pH = 6.0)) at 37 °C. (*) Data obtained in previous studies [15].

Alloys	E_OC_ (mV)	E_corr_ (mV)	J_corr_ (nA∙cm^−2^)	J_pass_ (μA∙cm^−2^)
CP Ti *	−383	−376	37.40	28.00
Ti25Ta25Nb3Sn *	−314	−249	1.72	12.71
Ti25Ta25Nb5Sn	−338	−341	16.00	82.95
